# Interannual variations of soil organic carbon fractions in unmanaged volcanic soils (Canary Islands, Spain)

**DOI:** 10.1002/ece3.355

**Published:** 2012-08-24

**Authors:** Cecilia María Armas-Herrera, Juan Luis Mora, Carmen Dolores Arbelo, Antonio Rodríguez-Rodríguez

**Affiliations:** 1 Departamento de Edafología y Geología, Universidad de La LagunaAvda. Astrofísico Francisco Sánchez, s/n, 38204, Tenerife, Islas Canarias, Spain; 2 Departamento de Ciencias Agrarias y del Medio Natural, Universidad de ZaragozaC/Miguel Servet, 177, 50013, Zaragoza, Spain

**Keywords:** Andosols, climate changes, litterfall, organic matter fractionation, root biomass, soil respiration, undisturbed ecosystems

## Abstract

The stability over time of the organic C stocked in soils under undisturbed ecosystems is poorly studied, despite being suitable for detecting changes related to climate fluctuations and global warming. Volcanic soils often show high organic C contents due to the stabilization of organic matter by short-range ordered minerals or Al-humus complexes. We investigated the dynamics of different organic C fractions in volcanic soils of protected natural ecosystems of the Canary Islands (Spain) to evaluate the stability of their C pools. The study was carried out in 10 plots, including both undisturbed and formerly disturbed ecosystems, over two annual periods. C inputs to (litterfall) and outputs from (respiration) the soil, root C stocks (0–30 cm), soil organic C (SOC) fractions belonging to C pools with different degrees of biogeochemical stability –total oxidisable C (TOC), microbial biomass C (MBC), water soluble C (WSC), hot-water extractable C (HWC), humic C (HSC), – and total soil N (TN) (at 0–15 and 15–30 cm) were measured seasonally.A statistically significant interannual increase in CO_2_ emissions and a decrease in the SOC, mainly at the expense of the most labile organic forms, were observed, while the root C stocks and litterfall inputs remained relatively constant over the study period. The observed changes may reflect an initial increase in SOC resulting from low soil respiration rates due to drought during the first year of study. The soils of nearly mature ecosystems were more apparently affected by C losses, while those undergoing the process of active natural regeneration exhibited disguised C loss because of the C sequestration trend that is characteristic of progressive ecological succession.

## Introduction

The C storage capacity of soils makes them a potentially important CO_2_ sink that play a role in the global C cycle. Soil organic C (SOC) is also one of the most active C pools, being involved in many processes related to the development and growth of plants and soil organisms and playing an active role in C fluxes and exchanges with the atmosphere, mainly in the forms of CO_2_ and CH_4_ (Janzen 2004; Lal 2005).

Numerous recent studies have reported variations in the contents of SOC at time scales usually ranging at least several decades (Rodeguiero et al. 2009). Several studies have addressed changes in SOC stocks over large areas or countries, in most of which the key role of land management is stressed and the effects of global warming are considered to be marginal (Smith et al. 2007; Van Wesemael et al. 2010). The effect of land use is also the focus of much work in experimental sites, but relations to environmental changes cannot be inferred from these studies unless the management had been unchanged for a period long enough to exclude its potential effect (Hopkins et al. 2009).

There has been much less attention to unmanaged soils, although they are seemingly suitable for investigating variations due to climate changes without interference from the effects of land management. SOC accumulation occurs during ecological succession until achieving a steady-state asymptote as the ecosystem approaches maturity and the C inputs are matched by C losses (Post and Kwon 2000). In mature ecosystems, SOC stocks are higher than in disturbed ecosystems (Lal 2005) and in equilibrium or quasi-equilibrium (Armas et al. 2007; Sierra et al. 2007), but disturbances and global warming may alter the balance between C influx and efflux, giving rise to disequilibrium and eventual change in SOC stocks (Trumbore 1997; Luo and Weng 2011).

**Figure 1:**
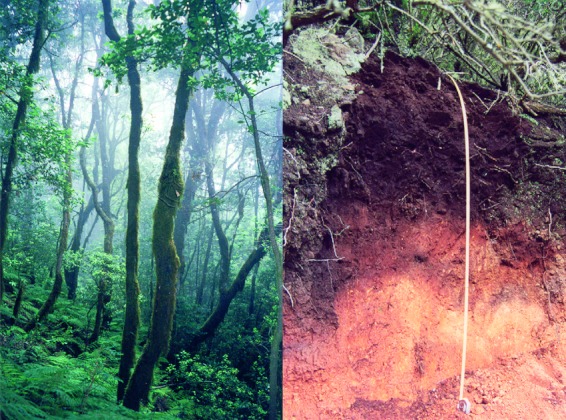
Organic-rich Andosols under laurel forest vegetation in the Canary Islands.

Soils of a volcanic origin, particularly Andosols (IUSS Working Group WRB 2006), are known for their ability to store large contents of organic (Fig. 1), which are attributed to the stabilization of soil organic matter in organo-metallic complexes, their association with short-range ordered minerals (allophane, imogolite, and ferrihydrite), and physical protection from microbial attack inside soil macroaggregates and microaggregates (Buurman et al. 2007; Tonneijck et al. 2010). Although C sequestration in Andosols is highly efficient, it is vulnerable both to erosion and aggregate breakdown (which might induce accelerated mineralisation of SOC) (Óskarsson et al. 2004; Rodríguez-Rodríguez et al. 2004, 2006; Mora Hernández et al. 2007).

Three main approaches for determining SOC stock changes with time are generally considered (Ellert et al. 2001). One of the most widely used is a repeated-inventory approach of SOC stocks at the same location over a sufficient period of time to detect statistically significant changes, which is estimated to be a minimum of 5–10 years (Smith 2004). Another approach is based on the analysis of all C fluxes entering and leaving the soil over a period of time, from which we can deduce the change trend in the stored SOC. C inputs to the soil are derived from decomposable plant materials, which are incorporated into the soil either from above-ground (litterfall) or from below-ground biomass (dead roots and root exudates). SOC losses result from decomposition and mineralization processes, which release CO_2_ and other gases, and to a lesser extent, from soil erosion and leaching. A third approach involves examining changes in specific fractions of C showing different turnover and soil residence times, which might reveal long-term trends in the evolution of SOC stocks (Denef et al. 2009).

In the context of global change, we considered that it would be of interest to carry out a study on the stability over time of SOC contents in undisturbed and formerly disturbed unmanaged volcanic soils of the Canary Islands (Spain). In this study, we investigated whether the SOC contents of volcanic soils are stable in the short term, or whether instead they may undergo consistent changes within short periods of time, such as a couple years. To this aim, we adopted a mixed approach involving a combination of the repeated-inventory, flux-analysis, and SOC fractions-based approaches. We studied changes in the main kinetic pools of SOC, as well as in the root C stock and the C fluxes into and out of the soil via litterfall and CO_2_ emissions as factors driving the long-term trends of SOC stocks. We also investigated whether such changes, if any, affect equally different types of soils and habitats, disturbed and nearly mature ecosystems, and both surface and subsurface horizons of the soils.

## Materials and Methods

### Study area and sampling design

**Figure 2:**
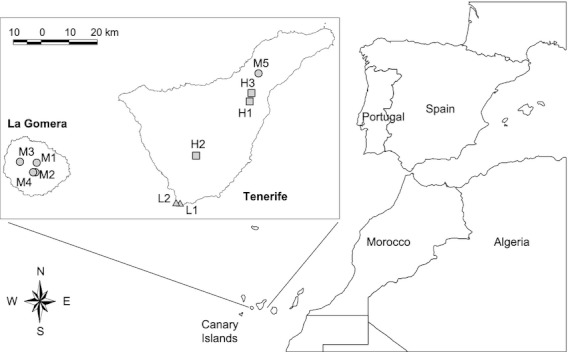
Location of the study sites.

The study was carried out in experimental plots located in natural ecosystems of the Tenerife and La Gomera Islands (Canary Islands, Spain) (Fig. 2). The natural ecosystems on the Canary Islands are distributed according to an altitudinal sequence in which three main habitats can be distinguished: arid lowland, humid midland, and xeric highland areas. The lowland areas are characterized by intense hydric and thermal stress conditions and vegetation that comprises xerophytic succulent scrubs. The midland areas are under the influence of humid trade winds, thus having favorable climate conditions that support the growth of subtropical laurel forests of high biodiversity and biomass. Finally, highland areas out of reach of the trade winds are characterized by moderate hydric and thermal stress and Canary pine (*Pinus canariensis*) forests (Fernández-Palacios and Nicolás 1995).

Our aim when setting the experimental plots was to select several samples that were representative of the soils and vegetation of the three main natural Canary Island habitats, including both nearly mature and human disturbed ecosystems. Using these criteria, 10 experimental sites were selected (Fig. 2), all of which remain unmanaged and located in protected natural areas: two in the lowland habitat (L1, L2), five in the midland (M1, M2, M3, M4, M5), and three in the highland (H1, H2, H3). Their general characteristics are summarized in Table 1 (see also Armas et al. 2007). L1, M1, M2, H1, and H2 are well-preserved samples of the original vegetation of these habitats: lowland succulent xerophytic scrubs (L1), midland broadleaved forests (M1, M2), and highland Canary pine forests (H1, H2) areas. In turn, L2, M3, M4, M5, and H3 host regressive plant communities resulting from past disturbances. L2 is a former dry-farming field located next to L1, abandoned several decades ago, and now hosting substitution scrubes. M3, M4, and M5 were disturbed by timber exploitation, cattle-herding, and wildfires in the past, but these disturbances ceased due to decreasing demand for fodder and fuel since the 1940s, and their eventual declaration as protected natural areas in the 1980s. Nowadays, the vegetation of these sites is comprised of degraded ericoid sclerophyllous forest (M3) and scrubland (M4) and a well-grown plantation of conifers carried out in mid-20th century (M5). Finally, H3 is a former Canary pine forest, deforested prior to the mid-20th century, whose current vegetation consists of native shrubby forage legumes.

**Table 1:** General characteristics of the experimental plots

Type of habitat	Plot code	Plant community	Dominant plant species	Soil classification (ISSS-ISRIC-FAO, 2006)
Lowland	L1	Mature *tabaiba* scrub	*Euphorbia balsamifera*, *E. canariensis*	Hypersalic Solonchaks
	L2	Regressive *salado* scrub	*Schizogyne sericea*, *Launaea arborescens*	Haplic Solonetzs
Midland	M1	Riparian laurel forest	*Persea indica*, *Laurus novocanariensis*	Aluandic eutrosilic fulvic Andosols
	M2	Laurel forest	*L. novocanariensis*, *Ilex canariensis*	Silandic eutrosilic fulvic Andosols
	M3	Degraded *Erica-Myrica* forest	*Erica arborea*, *Myrica faya*	Aluandic eutrosilic fulvic Andosols
	M4	Regressive shrubby heath	*E. arborea*, *Adenocarpus foliolosus*	Leptic Luvisols
	M5	Wood plantation	*Pinus radiata*, *Ilex canariensis*	Silandic fulvic Andosols
Highland	H1	Humid pine forest	*P. canariensis*, *E. arborea*	Luvic Phaeozems
	H2	Xeric pine forest	*P. canariensis*, *Lotus campylocladus*	Leptic Cambisols
	H3	Regressive broom scrub	*Chamaecytisus proliferus*, *Adenocarpus viscosus*	Silandic fulvic endoleptic Andosols

The main characteristics of the soils are shown in Table 2. In all cases, the soils have formed from basaltic tephra and scoriae resulting from the volcanic activity during the Quaternary. The andic character is very marked in all soils (qualifying as Andosols or not), but in the lowland soils, where the scarcity of soil organic matter (González-Pérez et al. 2007) hinders the stabilization of short-range minerals, which mostly evolve to crystalline forms.

**Table 2:** Main characteristics of the soils in the study plots

Profile	Horizon	Depth (cm)	Bulk density (Mg m^−3^)	pH (H_2_O)	EC_s_ (dS m^−1^)	CaCO_3_	TC	TOC	TN	Clay	Silt	Sand	Al_o_+½Fe_o_	P-retention (%)

						(g kg^−1^)								
L1	ABw	0–22	1.1	8.5	10.0	0.6	4.0	3.2	0.5	90	179	732	–	–
	Bw	22–35	1.1	8.1	29.2	2.0	3.9	2.5	0.3	76	229	695	–	–
	BwC	35–80	–	8.0	32.1	1.1	1.8	1.1	0.2	70	98	833	–	–
L2	BwA	0–22	1.3	9.3	3.4	1.7	3.5	1.9	0.3	94	209	697	–	–
	Bt	22–55	1.3	9.8	0.7	1.8	1.5	0.9	0.2	136	296	569	–	–
	BtC	55–70	1.4	10.0	0.8	1.0	1.2	0.8	0.2	166	211	623	–	–
M1	A1	5–25	0.4	5.7	–	–	128	139	10	176	522	303	20	89
	A2	25–70	0.7	6.3	–	–	47	44	4.6	277	492	231	27	90
	Bw	70– >120	–	6.1	–	–	22	19	4.1	248	507	245	–	–
M2	A	0–50	0.5	5.9	–	–	87	91	8.1	74	652	274	23	91
	Bw	50– >120	0.9	5.1	–	–	15	16	1.0	214	597	189	19	5.6
M3	ABw	0–50	0.7	5.9	–	–	127	128	8.9	97	448	454	20	86
	Bt	50– >100	0.8	5.7	–	–	61	52	2.8	647	226	127	23	89
M4	ABw	2–22	0.6	4.8	–	–	139	144	6.4	134	528	339	15	78
	Bt	22–35	0.7	4.8	–	–	71	76	4.8	219	509	272	18	90
M5	A1	7–26	0.4	6.0	–	–	113	114	7.0	97	561	342	45	95
	A2	26–72	0.5	5.8	–	–	63	64	5.0	84	668	246	58	96
	Bw	72– >120	0.5	5.8	–	–	24	19	2.0	110	487	403	76	94
H1	A	0–35	0.8	7.1	–	–	44	41	1.1	323	329	282	10	43
	Bt	35–100	0.8	7.1	–	–	6.5	5.6	0.4	403	382	180	12	45
H2	A1	0–5	1.1	5.7	–	–	69	59	7.2	127	415	459	5	34
	A2	5–10	1.1	5.8	–	–	45	42	4.0	175	461	363	5	35
	BwC	10–45	1.1	6.6	–	–	9.2	9.4	0.8	266	514	220	3	33
H3	A	0–15	0.8	5.2	–	–	122	124	11	69	347	584	22	81
	BtC	15–50/100	0.9	4.9	–	–	50	48	6.3	129	454	417	28	91

ECs, electrical conductivity in saturated extract; TC, total carbon; TOC, total organic (oxidisable) carbon; TN, total nitrogen; Al_o_, Fe_o_, acid-oxalate extractable Al and Fe; P-retention, phosphate retention.

**Figure 3:**
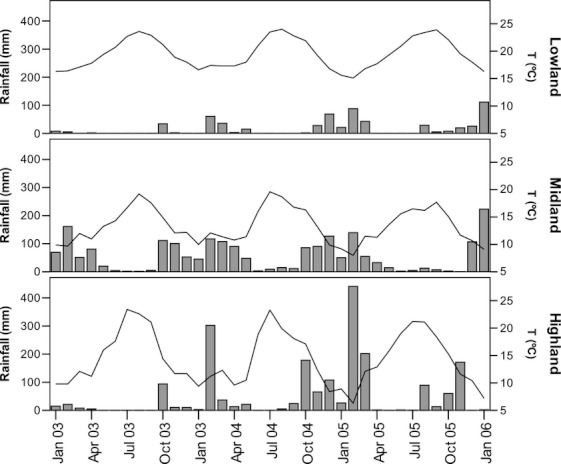
Rainfall and temperature at the experimental areas during the study period. Source: Cabildo de Tenerife (http://www.agrocabildo.org/agrometeorologia_estaciones.asp).

The size of the experimental plots was 25 × 25 m. Sampling was performed seasonally (spring, summer, autumn, and winter) in the same subplots (see below) over two annual periods separated by a 2-year interval: April 2003–January 2004 (Year 1), and April 2005–January 2006 (Year 2) (2 years × 4 seasons = 8 samplings). Figure 3 summarizes the meteorological conditions during the experimental period obtained from weather stations close to the sites L1-L2, M5, and H3. Temperatures varied only slightly between years. In contrast, rainfall was irregular, particularly in the lowland and highland areas, where the first year (2003) was significantly drier than the average for the decade 2000–2009 (*P* < 0.05, ANOVA [Analysis of Variance]).

### Analytical procedures

Various descriptive parameters of the soil C dynamics at the study sites were measured: litterfall C inputs, C-CO_2_ emissions from soil respiration, root C content (0–30 cm), SOC forms, and content and TN (at 0–15 and 15–30 cm).

#### Litterfall

To collect and quantify the litterfall in the forest ecosystems, a number of four permanent litter traps (53 × 53 cm) were placed at random in each experimental plot. Each trap was emptied and the litter was collected at the end of every season. In the arid habitat, litter traps are not suitable due to the shrubby size of the vegetation, and instead, four 1-m^2^ random subplots were delimited. Then, plant litter was removed from the soil surface, and litterfall residues were successively collected in each sampling period. Lignified (derived from woody tissues, e.g., twigs and bark) and nonlignified residues (mainly leaves, but also flowers and fruits) were separated. Samples were washed with deionized water, oven dried at 60°C to constant weight, and then weighed and pulverized. C content was determined using an elemental autoanalyzer (LECO, St. Joseph, MI), and C inputs from litter were calculated and expressed as g C m^−2^.

#### Soil respiration

The CO_2_ emitted from the soils was measured in the field using alkali trap static chambers following the method of Zibilske (1994). On each sampling date, three to four open-bottom steel chambers (dimensions 22 × 22 × 30 cm) were installed to a depth of 2 cm in the soil. Inside the chamber, a flask containing 50 mL of NaOH of a known concentration was placed on a tripod to minimize ground contact. A blank consisting of a closed-bottom steel chamber (dimensions 22 × 22 × 28 cm) containing the same alkaline solution of NaOH was also installed. All the chambers were hermetically sealed, and after 24 h, the alkaline solution was removed for analysis. NaOH content was determined using back-titration with a standard solution of HCl to the phenolphthalein end point, with previous addition of BaCl_2_ to facilitate carbonate precipitation. The results were expressed as mg C-CO_2_ m^−2^ h^−1^ (24 h).

#### Root carbon

At each site on each sampling date, two soil core samples were taken from a permanent 4 × 4 m subplot from the first 30 cm of topsoil using a 4 cm wide auger, air-dried and the roots were removed through a 0.5-mm mesh sieve. These roots samples were then washed with deionized water, oven dried at 60°C to constant weight, and finally, weighed and pulverized. C content was determined using an elemental autoanalyzer (LECO), and the results were expressed as kg C m^−2^ in the first 30 cm of soil.

#### SOC fractions and total N

For sampling soils, three subplots (4 × 4 m) were placed within each experimental plot. Soil cores (10 cm diameter) were collected within each subplot from 0 to 15 cm and 15–30 cm depths, and mixed to create an average sample. Soil samples were sieved through a 2-mm sieve and stored at 4°C until analysis. To evaluate distinct functional pools of organic matter with different turnover times, the following fractions were analyzed:

- Total organic C (TOC), using the Walkley and Black (1934) method. This method was preferred over high temperature combustion method due to the presence of significant amounts of carbonates in the soils of sites L1 and L2, and to the similarity of the values obtained using the two methods in the other soils (Table 2). Saline soil samples were treated with a silver sulphate solution to eliminate interference by chlorides during the analysis (Quinn and Salomon 1964).
- Microbial biomass C (MBC), using the chloroform-fumigation extraction procedure described by Vance et al. (1987) using a calibration factor of K_c_ = 0.38 to correct the efficiency of the extractive process.
- Water-soluble C (WSC), in 1:10 extracts obtained after 30 min of shaking, centrifugation at 1876 g and filtration through a nitrocellulose membrane filter with a pore size of 0.45 *μ*m (Ghani et al. 2003).
- Hot-water extractable C (HWC), according to the method described by Ghani et al. (2003) in 1:10 extracts (soil:water) obtained after the extraction of WSC, incubation at 80°C for 16 h, centrifugation at 1876 g, and filtration through a nitrocellulose membrane filter with a pore size of 0.45 *μ*m.
- Humic substances C (HSC), by 16 h of shaking with 0.1 M sodium pyrophosphate (Stevenson 1994) (proportion 1:100 soil:extractant), centrifugation at 15,316 g and filtration through MN 640 d filter paper (Macherey-Nagel; Düren, Germany). HSC content was corrected by subtracting the contents of WSC and HWC from the result obtained.
- Total N (TN), using the standard Kjeldahl method (Benton Jones 1991).

Microbial biomass C is considered to be the most important labile SOC and the main regulator of soil organic matter transformation and nutrient cycling, whereas WSC represents a very labile and highly mobile SOC fraction, HWC is widely used as a rough measure of the total labile C pool, and HSC might be representative of a more recalcitrant SOC pool (Von Lützow et al. 2007; Denef et al. 2009).

The results obtained were expressed on the basis of the dried-weight area (g m^−2^ or kg m^−2^), computed using the values of bulk density, coarse fragments, and soil water content. Bulk density was determined using drying at 105°C and weighing of soil cores samples of 250 cm^3^. Coarse fragments were determined using soil wet sieving. Soil water content was analyzed gravimetrically at 105°C.

#### Data analysis

The obtained results were analyzed in each study plot using a multifactorial ANOVA for main effects of time at intraannual (seasonal) and interannual scales and the depth of the soil samples and first-order interactions. The significance of the interannual variation was globally tested using repeated measures of ANOVA of the mean values at 0–30 cm depth in each plot, considering the differences between years as a within-subjects factor and the type of habitat as a between-subjects factor. Prior to the analyses, some variables were transformed to fulfill the normality and homoscedasticity requirements of the ANOVA. Post-hoc pairwise comparisons were carried out using the Tukey test. Statistical analyses were performed using the SPSS for Windows program (v.11.5; SPSS Inc., Chicago, IL).

In order to estimate the C balance at the ecosystem level, we calculated the average values of C influx via litterfall and efflux by soil respiration in each plot, extrapolated these values to a 2 years period, and compared them with the changes observed in the contents of C in the soil and plant roots at 0–30 cm depths during the study period. The results of the balance between C influx and efflux and of the variations in the C stocks should approach each other, following this formula:


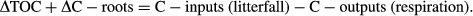


This simple model is only a rough estimation approach, as it is based on a very limited data set and on the assumptions that the changes in C contents mostly occurred in the topsoil layer (0–30 cm depth), litterfall was the major source of C supplies to the soil (root death and exudation are not considered), and soil respiration represented the only pathway of C loss from the soil (lixiviation and erosion are considered negligible).

## Results

### Litterfall

**Figure 4:**
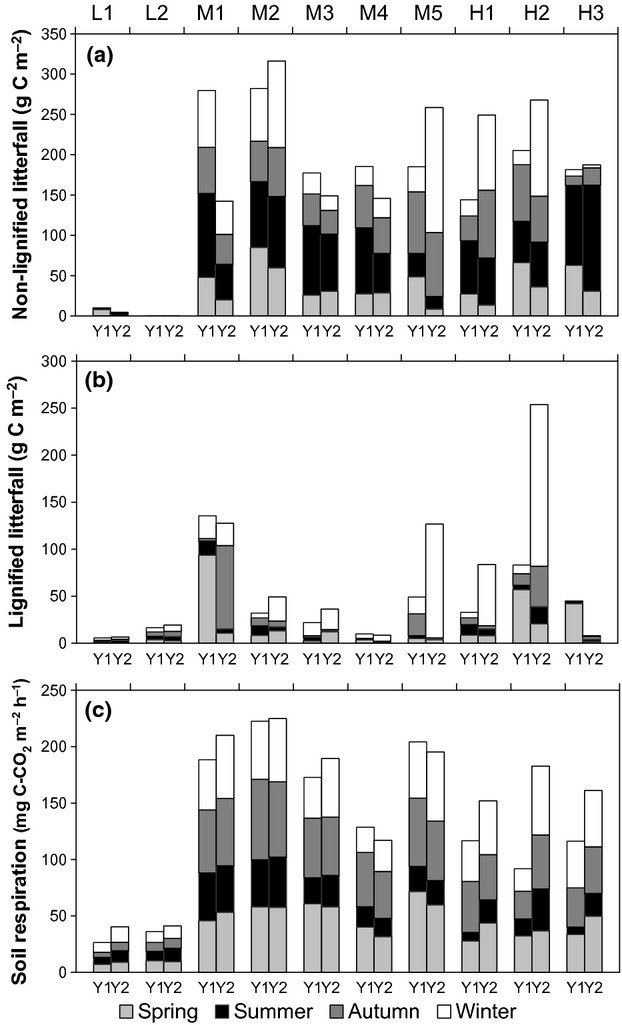
Soil carbon inputs (litterfall) and outputs (respiration) depending on sampling season, year and plot. (a) Litterfall unlignified residues (g C m^−2^); (b) Litterfall lignified residues (g C m^−2^); and (c) Soil respiration (mg C-CO_2_ m^−2^ h^−1^, 24 h). L1, Lowland climax scrub; L2, Lowland degraded scrub; M1 and M2, Midland climax forests; M3and M4, Midland degraded vegetation; M5, Midland afforestation; H1 and H2, Highland climax forests; H3, Highland degraded scrub; Y1, Year 1; Y2, Year 2.

The sum of the not-lignified (Fig. 4a) and lignified (Fig. 4b) litterfall inputs was greatest in the forests in the midland and highland areas (M1, M2, M5, H1, H2), intermediate in the midland and highland scrubs (M3, M4, H3), and least in the lowland scrubs (L1, L2). In the lowland habitat, litterfall was scarce and discontinuous and did not show a regular temporal pattern (*P* > 0.05, ANOVA). Lignified residues represented approximately half of the litterfall in deciduous-leaved, mature *tabaiba* scrub (L1), and almost all the input in the case of the reduced-leaved, nearly aphyllous, regressive *salado* scrub (L2). In midland and highland areas, the litterfall was mainly composed of unlignified residues. The input of leaves showed significant seasonal variations in four sites (M1, M3, M4, H3) with the largest inputs occurring in the summer, but showed significant interannual changes only in M1 (Table 3). The supply of twigs and branches was highly irregular in midland and highland areas and was influenced by the climate, being almost restricted to winter and spring, the seasons with the highest incidence of wind storms in the Canary Islands. With respect to this, it must be stressed that a rare tropical storm occurred in autumn of the second study year and severely affected the pine forest plots (M5, H1, H2).

**Table 3:** Analysis of variance (ANOVA) results of the litterfall C inputs and soil respiration in relation to the sampling season, year and plot

		L1	L2	M1	M2	M3	M4	M5	H1	H2	H3
Nonlignified litter C inputs	Season			*		**	**				*
	Year			**							
C-CO_2_	Season	*			*	*	*	*	*		**
	Year	**								*	*

** ns, not significant.

* Nonsignificant results are omitted.

**P < 0.01; *P < 0.05.

### Soil respiration

The highest rates of soil respiration were observed in midland forests (M1, M2, M3, M5), whereas the lowest were seen in arid lowland scrub communities (L1, L2) (Fig. 4c). At the ecosystem scale (plot mean values), soil respiration was found to correlate well with soil water content (Pearson correlation coefficient *r* = 0.760, *P* < 0.001) and litterfall inputs (*r* = 0.450, *P* < 0.001). We found significant seasonal variations in most sites (Table 3), the lowest respiration rates being observed in the summer in all cases except in L1, where we found the lowest values in the autumn. There was a very tight correlation (*r* = 0.867, *P* < 0.001) between the seasonal respiration rates in different years, so that they can be considered as highly representative of the season in each plot. Nevertheless, the soils of several plots (L1, H2, H3) significantly increased their respiration rates in the second year of the study.

### Root carbon

**Figure 5:**
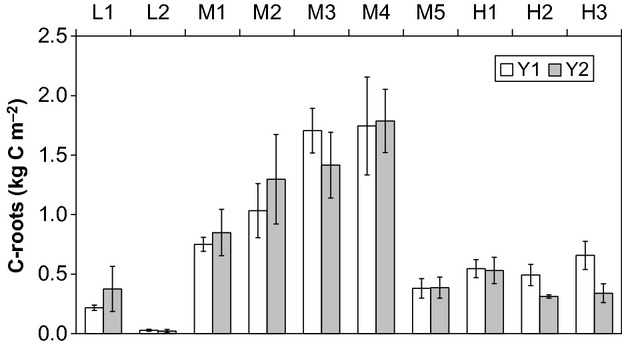
Root carbon content (kg m^−2^, 0–30 cm) depending on sampling year and plot. Values represent means ± SEM (between seasons). L1, Lowland climax scrub; L2, Lowland degraded scrub; M1 and M2, Midland climax forests; M3 and M4, Midland degraded vegetation; M5, Midland afforestation; H1 and H2, Highland climax forests; H3, Highland degraded scrub; Y1, Year 1; Y2, Year 2.

The root C content showed a high temporal stability (both seasonal and interannual) (*P* > 0.05, ANOVA) and pronounced differences depending on the habitat and degree of disturbance (Fig. 5). The greatest root C content occurred in soils of the midland areas, whereas it was intermediate in the highland and lowest in the lowland areas. The degraded sites in the lowland areas (L2) presented the lowest root C stocks, whereas those in the midland areas (M3, M4) showed the highest stocks; no apparent difference was observed between mature and regressive vegetation in the highland areas.

### Soil organic C and N

#### Differences between depths and locations

**Figure 6:**
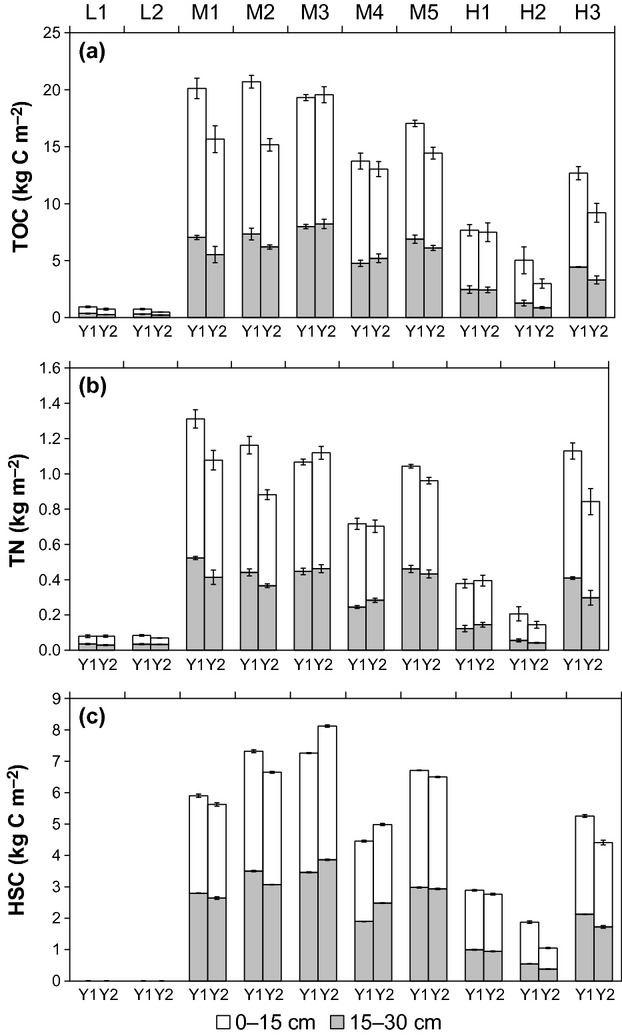
Contents of soil organic carbon, total nitrogen and humic carbon depending on sampling depth, year and plot. (a) Soil total organic carbon (TOC) (kg m^−2^); (b) Soil total nitrogen (TN) (kg m^−2^); and (c) Soil humic carbon (HSC) (kg m^−2^). Values represent means ± SEM (between seasons). L1, Lowland climax scrub; L2, Lowland degraded scrub; M1 and M2, Midland climax forests; M3 and M4, Midland degraded vegetation; M5, Midland afforestation; H1 and H2, Highland climax forests; H3, Highland degraded scrub; Y1, Year 1; Y2, Year 2.

**Figure 7:**
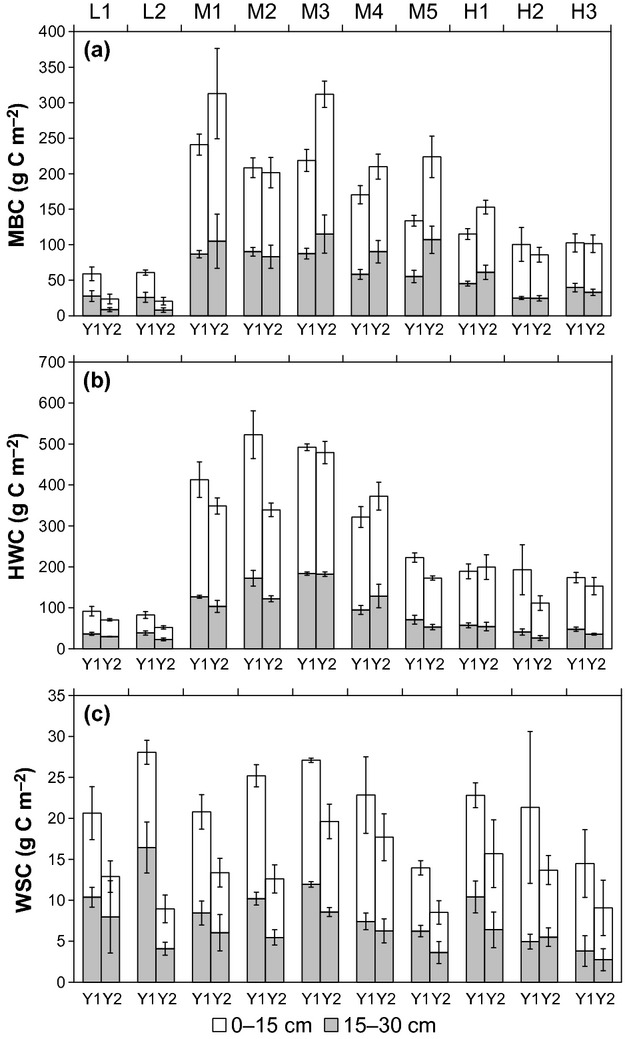
Contents of labile soil organic carbon fractions depending on sampling depth, year and plot. (a) Soil microbial biomass carbon (MBC) (g m^−2^); (b) Soil hot-water extractable carbon (HWC) (g m^−2^); and (c) Soil water-soluble C (WSC) (g m^−2^). Values represent means ± SEM (between seasons). L1, Lowland climax scrub; L2, Lowland degraded scrub; M1 and M2, Midland climax forests; M3 and M4, Midland degraded vegetation; M5, Midland afforestation; H1 and H2, Highland climax forests; H3, Highland degraded scrub; Y1, Year 1; Y2, Year 2.

All the SOC and N forms studied tended to be more abundant at 0–15 cm depths than at 15–30 cm depths (Figs. 6, 7), showing significant differences in most cases (Table 4). In almost all cases, depth showed no significant interactions with the seasonal and interannual variations, indicating that such variations affected both soil depths studied to a similar extent.

**Table 4:** Analysis of variance (ANOVA) results of the SOC and TN contents in relation to the sampling season, year, depth, and plot

		L1	L2	M1	M2	M3	M4	M5	H1	H2	H3
TOC	Year	*	*	*	*			*		**	**
	Depth	**	*	**	**	**	**	**	**	**	**
	Season × Year										*
TN	Season						**			*	*
	Year			**	**		*	*		*	**
	Depth		*	**	**	**	**	**	**	**	**
	Season × Year								**		**
	Season × Depth						**				
	Year × Depth						**				
HSC	Year	-	-			*	*			**	*
	Depth	-	-		*	*	*	**	**	**	**
MBC	Season	**									*
	Year	**	**			*		*	*		
	Depth			*	*	**	*		**	**	**
	Season × Year	*							*		*
HWC	Season						*				
	Year	*	*		*		*	*		*	
	Depth	*		**	**	**	**	**	**	**	**
	Season × Year					*					
WSC	Season				*		*			**	
	Year		*	*	**	*	*	*			
	Depth				**		**			**	**
	Season × Depth									*	

SOC, soil organic C; TOC, total organic carbon; TN, total nitrogen, HSC, humic C; MBC, microbial biomass C; HWC, hot-water extractable C; WSC, water soluble C.

Nonsignificant results are omitted.

** *P* < 0.01;

* *P* < 0.05.

The highest values of the SOC and N forms studied were mostly found in Andosols under natural forests in the midland area (M1, M2, M3), whereas they tended to be intermediate in the highland and lowest in the lowland areas. The HSC content was determined to be zero or not-different-from-zero in the soils of the lowland area (L1, L2). SOC and N levels exhibited the following approximate order: Andosols > Luvisols > Phaeozems > Cambisols > Solonetzs ≍ Solonchaks. The WSC content (Fig. 7c) is an exception, as it shows little variation among different types of habitats and soils.

### Seasonal variation

Total oxidisable C and HSC levels did not show significant seasonal patterns (Table 4). Labile SOC fractions (MBC, HWC, WSC) showed significant seasonal variations in only a few cases, resulting in erratic, highly irregular seasonal patterns over the study period. Significant seasonal variation was observed for TN in those sites (M4, H2, H3) where leguminous plants (*Adenocarpus foliolosus*, *A. viscosus*, *Chamaecytisus proliferus*, *Lotus campylocladus*) are abundant.

### Interannual variation

Soil organic C and N decreased (Table 4) in many of the sites studied, mainly at the expense of the most labile forms and, to a lesser extent, of humic forms. Particularly, TOC showed significant interannual changes in all, but three plots (Table 4, Fig. 6a). In the lowland area, an intense loss of TOC was observed both in the soils under mature (L1) and regressive (L2) scrubs, whereas TN (Table 4, Fig. 6b) was much less variable and did not show differences between years. In the midland area, the plots of greatest plant biomass (M1, M2, M5) showed notable interannual decreases of TOC and TN, whereas the soils of degraded sites (M3, M4) underwent significant increases of HSC (Table 4, Fig. 6c). With respect to the highlands, plot H2 showed a more significant decrease for TOC and HSC than for TN, whereas plot H3 exhibited a less pronounced decrease for HSC than for TOC and TN, and no significant differences were found for plot H1.

The MBC content showed a pronounced interannual decrease in the lowland plots (L1, L2), whereas in the midland areas, it did not significantly change under mature vegetation (M1, M2), but increased in certain disturbed plots (M3, M5) and significantly increased under humid pine forest (H1) in the highland zones (Table 4, Fig. 7a). The HWC content decreased in several plots (L1, L2, M2, M5, H2), increased only under shrubby heath (M4), and remained unchanged in the other plots (Table 4, Fig. 7b). In general terms, WSC content tended to decrease from the first to the second sampling year (Table 4, Fig. 7c), although the losses were not statistically significant in L1, H1, H2, and H3 due to the high variability in the observed values in these sites.

When we considered together the studied soils (Table 5), significant interannual decreases of TOC, HWC, and WSC were obtained affecting equally the three habitats considered (the interaction between the type of habitat and the interannual variation was not significant). Significant increase in the soil respiration rates was also observed affecting particularly the lowland and highland sites, whereas root C stocks, litterfall inputs and TN, HSC, and MBC levels showed no consistent interannual variations.

**Table 5:** Interannual variation (%) of the C fluxes and pools and TN of the soils studied in relation to the type of habitat

	C-litterfall	C-CO_2_	C-roots	TOC	TN	HSC	MBC	HWC	WSC
Lowland	−5.3	+30.6	+61.3	−26.4	−6.9	−	−61.9	−29.0	−50.0
Midland	+0.3	+2.2	+2.2	−13.5	−9.7	+0.7	+30.9	−11.3	−36.8
Highland	+51.7	+53.0	−30.3	−24.0	−20.7	−18.8	+3.9	−18.5	−35.7
Total	+52.0	+34.2	+14.4	−17.4	−9.4	−5.8	+10.3	−12.6	−29.2
ANOVA									
Year		**		**				*	**
Habitat	**	**	*	**	**	**	**	**	
Year × Habitat		*					*		

TOC, total organic carbon; TN, total nitrogen, HSC, humic C; MBC, microbial biomass C; HWC, hot-water extractable C; WSC, water soluble C; ns, not significant.

Nonsignificant results are omitted.

** *P* < 0.01;

* *P* < 0.05.

### Discussion

#### Differences between sites

**Figure 8:**
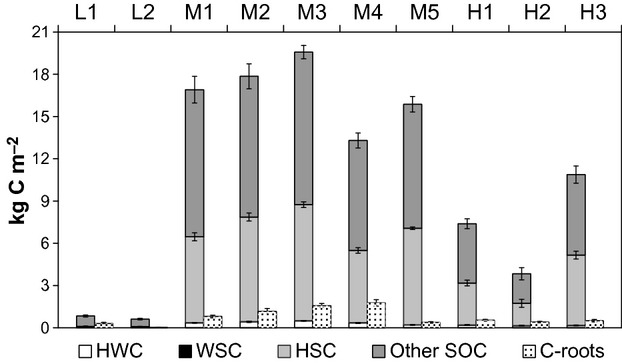
Average contents of the main organic carbon pools in soils and plant roots (kg m^−2^, 0–30 cm) depending on the plot. Values represent means ± SEM (between seasons). HWC, Soil hot-water extractable carbon; WSC, Soil water-soluble carbon; HSC, Soil humic carbon; Other SOC, other soil organic carbon; C-roots, Root carbon content; L1, Lowland climax scrub; L2, Lowland degraded scrub; M1 and M2, Midland climax forests; M3 and M4, Midland degraded vegetation; M5, Midland afforestation; H1 and H2, Highland climax forests; H3, Highland degraded scrub.

Figure 8 summarizes the relative contribution of each of the main C pools to the total C stock in the different ecosystems. The lowland ecosystem presented the soils with the lowest organic content due to the aridity and scarce and discontinuous litterfall supplies in these areas. The degree of humification at these sites was found to be low and reflects poorly transformed organic matter, which is typical of soils in these areas (González-Pérez et al. 2007). In the lowland areas, SOC contents were lower in the disturbed than in the undisturbed site probably because of the predominance of lignified residues in the litter and the lesser root development of the regressive vegetation.

Higher SOC levels were found in the midland and highland as a result of a more humid climate and higher organic inputs than in the lowland areas. Andosols exhibited the highest values of TOC, TN, and HSC presumably due to the stabilization of these SOC pools through the formation of complexes with short-range-ordered minerals, which are characteristic in these soils. The levels of TOC, TN, and HSC in Andosols under mature forests (M1, M2) were close to those under late-successional secondary forest (M3), and only slightly higher than those under regressive scrub (H3), suggesting that the degree of ecosystem maturity plays only a minor role in the SOC contents in soils of the midland and highland areas. This result is consistent with the reported by Torn et al. (1997) that C sequestration in Andosols is active for a longer time than in other soil types, long after the ecosystem reaches maturity, so SOC stocks may differ due to the differing SOC accumulation history during soil formation rather than to shorter-term successional changes.

Regarding the SOC labile fractions, MBC and HWC depended mainly on the habitat type: the levels of MBC and HWC were remarkably lower under pine forests (M5, H1, H2) than under broadleaved forest (M1, M2, M3), probably because of the sclerophylly of pine needle litter, which results in slower decompositions rates and lower inputs of labile organic forms (Coûteaux et al. 1995). This pattern was not observed, however, for WSC levels, which were similar in the soils of all the habitats studied.

#### Temporal variations

The results show significant changes in SOC contents in most soils studied after a short period of time, of only 2 years. We consider such variations as severe, especially those observed in nearly mature ecosystems where the contents of SOC are assumed nearly constant. The variations affected particularly the more labile fractions (WSC, HWC), but also the total organic C (TOC) and, to a lesser extent, slowly oxidisable fractions (HSC), in spite of the low intraannual variability shown by these last two pools.

Changes in SOC contents were not homogeneous, but had different and sometimes divergent behavior depending on the type of habitat and soil and past disturbances. The SOC loss was not apparent in soils of plots M3 and M4, where there was instead an increase in humic C. Our interpretation is that the SOC losses in these sites may have been masked by the tendency to sequester C that is characteristic of progressive ecological succession. The decrease in the contents of the labile fractions studied, which have a low relative contribution to the total SOC, are not sufficient to explain the observed reduction in TOC. This result necessarily implies a decrease in the content of other labile fractions not considered in this study, like light or particulate organic C (POC), which is a major component of labile SOC pool along with MBC and WSC (García et al. 2005).

The observed interannual changes were equally intense in both depths of study considered. This finding adds to growing evidence that contemporary changes in the SOC contents may affect a considerable thickness of soil (Meersmans et al. 2009), so that studies of the dynamics of SOC stocks should not be limited to the study of topsoil. Although the need to account for the subsoil for estimating the SOC stocks has been stressed (VandenBygaart and Angers 2006; Don et al. 2007), dynamic studies concerning SOC are still focused on the very topsoil, which is considered much more variable and easy to change (Tan et al. 2004; Viglizzo et al. 2011).

Obviously, the changes reported in this study cannot be considered a trend, but the evidence of significant oscillations of the SOC for short periods of time. Several studies based on the measurement of C fluxes (Dunn et al. 2007; Allard et al. 2008; Koehler et al. 2011) have recorded significant interannual variations in the amount and sign of the C exchanges to or from the soils, so the same soils may act as sinks or as issuers of C in subsequent years, regardless of whether the longer-term trend is to remain stable or to change. These oscillations, which could affect the soil C budget, were attributed to climate variability. Patterns of variation depending on the type of habitat and the degree of human disturbance at regional scale were also found by Yang et al. (2009) and interpreted as possible different responses to climate warming.

As neither the stock of roots nor the litterfall inputs showed interannual decreases, the loss of SOC underwent by the studied soils should be related to the higher respiratory rates recorded in the second year, particularly in the lowland and highland ecosystems. The year 2003 was especially warm and dry throughout this region of the world (Brunet et al. 2007), and significantly dry in the lowland and highland study areas. Soil water content was decisive in respiratory rates during the study period, and is generally considered key to soil respiration in water-limited habitats (Conant et al. 2004). An unusually severe drought during the first year of the study could have caused a temporary accumulation of SOC, which in otherwise normal conditions would have been mineralized. Borken et al. (2006) already described the transient behavior of a soil as a C sink, as a result of experimentally induced drought.

Nevertheless, the observed loss of TOC is much greater (about 10 times greater) than that deduced from extrapolating the annual balance between the mean litter inputs and emissions of CO_2_ to the entire study period. This imbalance could be explained either by an exaggerated underestimation of the respiration rates due to the method used (Hernández Fernández and García Izquierdo 2003) or by the loss of important amounts of SOC through mechanisms other than CO_2_ emissions, such as soil erosion. An important contribution of erosion to SOC losses is consistent with the comparable losses of C and N observed in several of the soils studied, particularly in Andosols. However, in certain nonandic soils, only C losses are significative, which indicates a predominance of selective loss of C due to mineralization. This finding highlights the importance of Andosols as stable C sinks and the role of soil erosion as a key process in the loss of SOC stocked in Andosols.

We found interannual variations on C balance at the ecosystem scale that, when observed, can be mostly explained by climatic fluctuations from 1 year to another. Such sensitivity to climate variations is likely to lead to very rapid and severe changes on C balance at the ecosystem level in response to consistent changes in climate. Anyway, our findings should warn us to be cautious when analyzing SOC changes reported in short- or mid-term follow-up, as the sequence of a few years of deviation from average climate conditions is sufficient to produce a significant drift on SOC contents.
